# Multiple Gastrointestinal Immune-Related Adverse Events From Immune Checkpoint Inhibitor Therapy

**DOI:** 10.14309/ctg.0000000000000768

**Published:** 2024-09-12

**Authors:** Trevor S. Barlowe, Shruti Saxena-Beem, Rumey C. Ishizawar, Hans Herfarth, Andrew M. Moon

**Affiliations:** 1 Division of Gastroenterology and Hepatology, Department of Medicine, University of North Carolina School of Medicine, Chapel Hill, North Carolina, USA;; 2 Division of Rheumatology, Department of Medicine, University of North Carolina School of Medicine, Chapel Hill, North Carolina, USA;; 3 Lineberger Comprehensive Cancer Center, University of North Carolina School of Medicine, Chapel Hill, North Carolina, USA.

**Keywords:** colitis, enteritis, hepatitis, pancreatitis

## Abstract

**INTRODUCTION:**

We aimed to describe immune-related adverse events (irAEs) affecting multiple organs of the gastrointestinal system in patients who received immune checkpoint inhibitors.

**METHODS:**

Within a 2,843 patient retrospective cohort consisting of patients with cancer treated with immune checkpoint inhibitors, we used the Electronic Medical Record Search Engine, an information retrieval system, to search free text in the medical record to identify patients with multiple gastrointestinal irAEs.

**RESULTS:**

Thirteen patients developed multiple gastrointestinal irAEs (0.46%). The most common patterns of multisystem gastrointestinal irAE were colitis + pancreatitis and colitis + enteritis.

**DISCUSSION:**

Multisystem gastrointestinal irAEs are rare but warrant further characterization and attention.

## INTRODUCTION

Immune checkpoint inhibitors (ICIs) are cancer therapies that activate the host immune response by blocking immune suppressive signaling pathways, such as cytotoxic T-lymphocyte antigen (CTLA-4), programmed cell death 1 (PD-1), and ligand (1). ICI use is increasing, and up to 40% of patients with newly diagnosed malignancy may now be eligible for ICI therapy (2).

Inflammatory side effects, termed immune-related adverse events (irAEs), are serious side effects of ICIs that lead to disruption or discontinuation of therapy. Much of the existing literature and clinical trial data categorize irAEs as single-organ events (3). Single-organ gastrointestinal irAEs, such as colitis and hepatitis, are common and have been well described (4,5). However, there is emerging evidence that irAEs can impact multiple organs at once, termed multisystem irAEs, which remain poorly characterized (3). The incidence of multisystem irAEs has been reported between 5% and 40% (6–9) and varies depending on ICI regimen and malignancy. However, there are clear patterns of co-occurring irAEs (6,10). Specific evaluation of multisystem irAEs involving organs of the gastrointestinal system could provide important guidance for gastroenterology and hepatology providers who will encounter increasing irAEs in the future.

We therefore aimed to describe multisystem irAEs affecting organs of the gastrointestinal system, using a text-based information retrieval system to search unstructured clinical data, such as clinical notes or pathology reports, within a retrospective cohort of patients with cancer who received ICI.

## METHODS

A 2,843 patient cohort was identified consisting of all patients with cancer who received 1 or more dose of any single or combination of ICI (including ipilimumab, nivolumab, pembrolizumab, atezolizumab, and durvalumab) within the University of North Carolina healthcare system between 2014 and 2019 (11). We then used the Electronic Medical Record Search Engine, a validated information retrieval system (12,13), to search free text (unstructured) clinical data in the medical record to identify patients within this cohort who developed multiple gastrointestinal irAEs. The search included any 2 of the most common GI irAEs (“colitis,” “hepatitis,” “pancreatitis,” “enteritis,” “duodenitis,” “esophagitis,” “gastritis”), and terms that implied immune origin (“irAE,” “auto∼immune,” “immune∼mediated”). The full search is available in the Supplemental Information (see Supplementary Digital Content 1, http://links.lww.com/CTG/B203).

The search identified 268 patients. A chart review in the medical record was performed to abstract clinical data. irAEs were determined according to available diagnostic criteria (4,5). We defined irAE as (i) clear documentation of irAE in the chart and (ii) at least 1 form of objective evidence of irAE, such as laboratory testing, imaging, pathology, endoscopy, or physical examination findings. For ICI pancreatitis, given the poor specificity of lipase elevation in the setting of luminal inflammation, we required 2 forms of objective evidence of irAE. We included patients who had simultaneous and sequential development of multiple irAE per available guidelines (3).

The primary outcome of this descriptive study was to estimate the prevalence of multisystem gastrointestinal irAE and to evaluate patterns of multisystem gastrointestinal irAE. We collected covariate data on age, sex, race, cancer and ICI factors, and irAE factors. Univariate statistics were used to describe covariate distribution among identified patients. This study was approved by the University of North Carolina Institutional Review Board.

## RESULTS

Of the 2,843 patients in our cohort, we identified 13 patients who developed multiple gastrointestinal irAEs (0.46%). Clinical characteristics of the identified patients are summarized in Tables 1 and 2. Almost 50% of identified patients received ICI to treat metastatic melanoma. ICI regimens included anti PD1, anti CTLA-4, or combination of anti PD1/anti CTLA-4 for a varying number of cycles (4.0 ± 10.3). Three of 13 patients had progression of their malignancy while being treated with ICI.

**Table 1.** Summary of patients who developed multiple gastrointestinal irAEs

Characteristic	N = 13
Age, yr, mean ± SD	46.4 ± 18.7
Female sex, n (%)	10 (76.9)
Race, n (%)	
White	12 (92.3)
Other	1 (7.7)
Type of malignancy, n (%)	
Melanoma	6 (46.1)
Lung adenocarcinoma	2 (15.4)
Colon adenocarcinoma	2 (15.4)
Renal cell carcinoma	2 (15.4)
Hodgkin lymphoma	1 (7.7)
ICI regimen, n (%)	
Pembrolizumab	4 (30.8)
Nivolumab	3 (23.1)
Ipilimumab	3 (23.1)
Ipilimumab/nivolumab combination	3 (23.1)
Cycles of ICI before GI irAE, mean ± SD	4.0 ± 10.3
Time to development GI irAE, d, mean ± SD	72.0 ± 326.0
Multisystem GI irAE, n (%)^a^	
Colitis + pancreatitis	4 (30.8)
Colitis + enteritis	4 (30.8)
Gastritis + enteritis	3 (23.1)
Colitis + gastritis	2 (14.4)
Colitis + hepatitis	2 (14.4)
Hepatitis + pancreatitis	2 (14.4)
Enteritis + pancreatitis	1 (14.4)
Gastritis + pancreatitis	1 (14.4)
Associated irAE in other organ systems, n (%)^b^	
Rheumatologic/musculoskeletal (PMR, arthritis, myositis)	6 (46.1)
Endocrine (thyroid, diabetes, adrenal insufficiency)	3 (23.1)
Dermatologic (rash)	2 (14.4)
Cardiac (myocarditis)	1 (7.7)
Neurologic (myasthenia)	1 (7.7)
GI irAE treatment, n (%)	
Steroids	13 (100)
Infliximab	4 (30.8)
Vedolizumab	3 (23.1)
Mycophenolate	1 (7.7)
GI irAE responded to treatment, n (%)	12 (92.3)
Cancer response to ICI, n (%)	
Response (complete or partial)	4 (30.8)
Stable	6 (46.1)
Progression	3 (23.1)

EMERSE, Electronic Medical Record Search Engine; GI irAE, gastrointestinal system-specific immune-related adverse event; ICI, immune checkpoint inhibitor; irAE, immune-related adverse event; PMR, polymyalgia rheumatica.

a In patients with 3 organs involved, all organ combinations were reported.

b irAEs in other organ systems were identified during a chart review but not systematically searched for in the cohort using EMERSE.

**Table 2.** Clinical characteristics of 13 patients with multiple gastrointestinal irAEs

ID	Age	Sex	Race/ethnicity	Primary malignancy	ICI therapy	ICI cycles^a^	Cancer response to ICI	GI irAE	Other irAE	Reason for ICI d/c^b^	GI irAE treatment^c^	GI irAE response^d^
1	70	Male	White	Renal cell carcinoma	Nivolumab	38	Progression	Colitis, pancreatitis	PMR	Pancreatitis	Steroids	Y
2	48	Female	White	Colon adenocarcinoma	Ipilimumab/nivolumab combination	3	Stable	Colitis, pancreatitis	Adrenal insufficiency	Pancreatitis	Steroids	Y
3	26	Female	Other	Hodgkin lymphoma	Nivolumab	18	Stable	Colitis, pancreatitis	—	Pancreatitis	Steroids, vedolizumab	Y
4	40	Male	White	Melanoma	Nivolumab	5	Response	Enteritis, gastritis, pancreatitis	Inflammatory arthritis	Pancreatitis	Steroids, infliximab	Y
5	48	Female	White	Lung adenocarcinoma	Pembrolizumab	6	Stable	Colitis, pancreatitis	Rash, inflammatory arthritis, hypothyroid	Inflammatory arthritis	Steroids	Y
6	46	Female	White	Melanoma	Ipilimumab	2	Stable	Colitis, enteritis	—	Colitis	Steroids, infliximab, vedolizumab	Y
7	21	Female	White	Melanoma	Ipilimumab/nivolumab combination	1	Progression	Colitis, gastritis, enteritis	Hypothyroid	Colitis	Steroids, infliximab	Y
8	72	Female	White	Cecal adenocarcinoma	Pembrolizumab	1	Stable	Colitis, hepatitis	Myasthenia gravis, myositis, myocarditis	Combination of irAE	Steroids	N^e^
9	58	Female	White	Melanoma	Ipilimumab	2	Progression	Colitis, gastritis, enteritis	—	Combination of irAE	Steroids, infliximab	Y
10	80	Female	White	Lung adenocarcinoma	Pembrolizumab	5	Response	Colitis, hepatitis	—	Combination of irAE	Steroids	Y
11	47	Male	White	Melanoma	Ipilimumab	2	Stable	Hepatitis, pancreatitis	Inflammatory arthritis, worsened psoriasis	Combination of irAE	Steroids, mycophenolate	Y
12	67	Female	White	Renal cell carcinoma	Pembrolizumab	3	Response	Hepatitis, pancreatitis	Diabetes, inflammatory arthritis	Combination of irAE	Steroids	Y
13	27	Female	White	Melanoma	Ipilimumab/nivolumab combination	9	Response	Colitis, enteritis	—	Colitis	Steroids, vedolizumab	Y

d/c, discontinuation; GI irAE, gastrointestinal system-specific immune-related adverse event; ICI, immune checkpoint inhibitor; irAE, immune-related adverse event; other irAE, immune-related adverse event occurring in any other organ system aside from GI; PMR, polymyalgia rheumatica.

a Total number of cycles of ICI before discontinuation.

b Reason cited in the medical record for why ICI was discontinued.

c All treatments received by patients for treatment of gastrointestinal irAE.

d Eventual response to therapy for gastrointestinal irAE, yes or no (Y/N).

e Patient developed fatal multisystem irAEs.

The most common patterns of multisystem gastrointestinal irAE were colitis + pancreatitis and colitis + enteritis. Six of 11 patients with colitis required treatment with biologics due to failure of steroids to control symptoms. One of 4 patients with hepatitis required mycophenolate. All other gastrointestinal irAEs responded to steroids, aside from 1 patient who developed fatal multisystem irAE including myasthenia and myocarditis.

**Figure 1.**
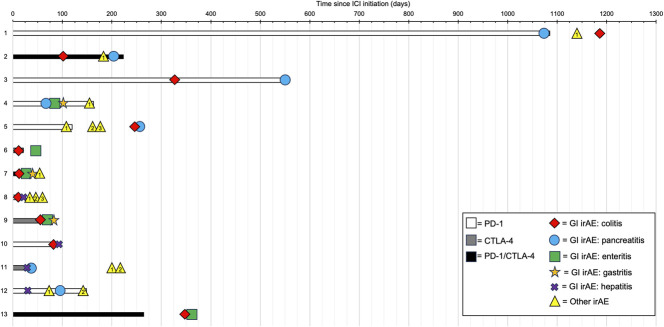
Swimmer plot illustrating patients treated with ICI who developed multiple gastrointestinal irAEs. GI irAE, gastrointestinal system-specific immune-related adverse event; ICI, immune checkpoint inhibitor; other irAE, immune-related adverse event occurring in any other organ system aside from GI. Other irAEs: patient 1: 1 = PMR; patient 2: 1 = adrenal insufficiency; patient 4: 1 = inflammatory arthritis; patient 5: 1 = inflammatory arthritis, 2 = rash, 3 = hypothyroidism; patient 7: 1 = hypothyroidism, patient 8: 1 = myasthenia gravis, 2 = myositis, 3 = myocarditis; patient 11: 1 = inflammatory arthritis, 2 = worsened psoriasis; patient 12: 1 = diabetes, 2 = inflammatory arthritis.

The timing of irAE development is shown in Figure 1. There was no consistent temporal relationship between ICI administration, gastrointestinal irAEs, or irAEs in other organ systems. The mean number of days to first GI irAE varied widely (72.0 ± 326.0).

## DISCUSSION

This study evaluated a retrospective cohort of patients who received ICI and developed multisystem gastrointestinal irAEs. Using a stringent case definition of irAE, we found that multisystem gastrointestinal irAEs are rare, with a prevalence of 0.45%. The most common multisystem gastrointestinal irAEs were colitis + pancreatitis and colitis + enteritis.

The prevalence and patterns of multisystem gastrointestinal irAE have not previously been reported. Patients with pan-luminal irAE have been described, with studies finding that 50%–70% of patients with upper gastrointestinal tract irAE, such as gastritis, have concomitant ICI enteritis/colitis (14,15). Our study, which evaluated all organs in the digestive system, adds novel information to the current understanding of multisystem irAEs.

As use of ICIs increase, characterization of irAE, including multisystem events, will be important for clinical and research efforts. Practitioners who care for patients with irAE should diligently evaluate for systemic toxicities and be aware of the patterns of multisystem involvement identified in this study, such as colitis + pancreatitis and colitis + enteritis. Furthermore, the patients described in this study represent a severe class of irAE, with over half being steroid resistant or nonresponsive. Involvement of multiple organs creates challenging treatment decisions for which multidisciplinary approaches are needed and no current treatment guidelines exist. Our study provides preliminary evidence that standard single-organ irAE treatments can be effective for multisystem gastrointestinal irAE.

This study has limitations. Data were collected from a single health system. The case finding protocol was limited to information included in free text portions of the medical record. Finally, comparative statistical analyses were not performed in this descriptive study due to the heterogenous nature of identified patients.

In conclusion, this study describes the clinical characteristics of patients who developed multiple gastrointestinal irAEs, estimated a prevalence of multisystem gastrointestinal irAE of 0.45% and identified patterns of multisystem gastrointestinal irAE including colitis + pancreatitis and colitis + enteritis.

## CONFLICTS OF INTEREST

**Guarantor of the article:** Trevor S. Barlowe, MD.

**Specific author contributions:** T.S.B.: conceptualization, methodology, formal analysis, investigation, visualization, writing—original draft. S.S.-B., R.C.I., and H.H.: conceptualization, methodology, writing—review and editing. A.M.M.: conceptualization, methodology, supervision, writing—review and editing.

**Financial support:** T.S.B. was supported by a grant from the National Institutes of Health (T32 DK007634). A.M.M. was supported by a Clinical, Translational and Outcomes Award (CTORA) from the AASLD Foundation. This study was supported by the UNC Thurston Arthritis Research Center Dean's Fund. i2b2, EMERSE, were Carolina Data Warehouse for Health were used in conducting this study. These research tools at the University of North Carolina are supported by the National Center for Advancing Translational Sciences (NCATS), National Institutes of Health, through Grant Award Number UM1TR004406. The content is solely the responsibility of the authors and does not necessarily represent the official views of the NIH.

**Potential competing interests:** T.S.B., S.S.-B., and R.C.I.: no disclosures. H.H.: consultant or advisory board for Alivio, AMAG, BMS, Boehringer, Celltrion, ExeGI, Finch, Fresenius Kabi, Galapagos, Gilead, Janssen, Lycera, Merck, Otsuka, Pfizer, PureTech, Seres, Ventyx and research support from Artizan Biosciences, Allakos, Novo Nordisk, and Pfizer. A.M.M.: consulting for TARGET RWE, DSMB for Intercept Pharmaceuticals, research funding (to institution) from DCN diagnostics.

## Supplementary Material
