# Supplementary material for: Multiple Gastrointestinal Immune-Related Adverse Events From Immune Checkpoint Inhibitor Therapy
Source: Clin Transl Gastroenterol. 2024 Sep 12;15(11):e00768. doi: 10.14309/ctg.0000000000000768 (PMC11596359; doi:10.14309/ctg.0000000000000768)
Supplement: Supplementary file 2 [file ct9-15-e00768-s002.docx]

**Supplemental Information**

EMERSE search term:

(("colitis" AND "pancreatitis") OR ("colitis" AND "hepatitis") OR ("colitis" AND "enteritis") OR ("colitis" AND "duodenitis") OR ("colitis" AND "gastritis") OR ("colitis" AND "esophagitis") OR ("pancreatitis" AND "hepatitis") OR ("pancreatitis" AND "enteritis") OR ("pancreatitis" AND "duodenitis") OR ("pancreatitis" AND "gastritis") OR ("pancreatitis" AND "esophagitis") OR ("hepatitis" AND "enteritis") OR ("hepatitis" AND "duodenitis") OR ("hepatitis" AND "gastritis") OR ("hepatitis" AND "esophagitis") OR ("enteritis" AND "duodenitis") OR ("enteritis" AND "gastritis") OR ("enteritis" AND "esophagitis") OR ("duodenitis" AND "gastritis") OR ("duodenitis" AND "esophagitis") OR ("gastritis" AND "esophagitis")) AND ("IRAE" OR "auto~immune" OR "immune~mediated")
